# Cardiovascular Risk in Philadelphia-Negative Myeloproliferative Neoplasms: Mechanisms and Implications—A Narrative Review

**DOI:** 10.3390/cimb46080496

**Published:** 2024-08-02

**Authors:** Samuel Bogdan Todor, Cristian Ichim, Adrian Boicean, Romeo Gabriel Mihaila

**Affiliations:** Faculty of Medicine, Lucian Blaga University of Sibiu, 550169 Sibiu, Romania; samuelbogdant@gmail.com (S.B.T.); romeo.mihaila@ulbsibiu.ro (R.G.M.)

**Keywords:** myeloproliferative neoplasms, cytokines dysregulations, JAK 2 mutation, essential thrombocythemia, polycythemia vera, primary myelofibrosis

## Abstract

Myeloproliferative neoplasms (MPNs), encompassing disorders like polycythemia vera (PV), essential thrombocythemia (ET), and primary myelofibrosis (PMF), are characterized by clonal hematopoiesis without the Philadelphia chromosome. The JAK2 V617F mutation is prevalent in PV, ET, and PMF, while mutations in MPL and CALR also play significant roles. These conditions predispose patients to thrombotic events, with PMF exhibiting the lowest survival among MPNs. Chronic inflammation, driven by cytokine release from aberrant leukocytes and platelets, amplifies cardiovascular risk through various mechanisms, including atherosclerosis and vascular remodeling. Additionally, MPN-related complications like pulmonary hypertension and cardiac fibrosis contribute to cardiovascular morbidity and mortality. This review consolidates recent research on MPNs’ cardiovascular implications, emphasizing thrombotic risk, chronic inflammation, and vascular stiffness. Understanding these associations is crucial for developing targeted therapies and improving outcomes in MPN patients.

## 1. Introduction

Myeloproliferative neoplasms (MPNs) are malignant hematologic diseases characterized by the clonal production of hematopoietic cells. These disorders encompass polycythemia vera (PV), essential thrombocythemia (ET), and primary myelofibrosis (PMF) [1]. They share a common characteristic in that they lack the Philadelphia chromosome [2]. The overall age-standardized incidence of MPNs was found to be 4.45 cases per 100,000 person-years for all MPNs combined. Specifically, the incidence rates were 1.48 for PV, 1.60 for ET, and 0.53 for PMF, with higher rates among older age groups [3]. The occurrence of the JAK2 V617F (Janus Kinase 2) mutation is notably high, up to 97% in individuals with PV and approximately 60% in those with ET and PMF, with no significant difference between prefibrotic and overt PMF [4,5,6,7]. Furthermore, anomalies in the thrombopoietin receptor MPL are observed in 5% of patients with ET and PMF but are absent in patients with PV [8,9]. For the majority of ET and PMF patients without mutations in JAK2 or MPL, Calreticulin (CALR) variants are prevalent, occurring in 25% of cases [10,11]. However, some patients are classified as triple-negative MPN, meaning they lack any of the genetic anomalies. In these cases, epigenetic regulators (such as TET2, ASXL1), splicing factors, and DNA repair proteins (such as tumor protein 53). Importantly, these alterations are also found in different types of myeloid neoplasms [11]. Patients with these conditions are susceptible to leukemic transformation or the development of myelofibrosis [12]. Essential thrombocytosis is generally a slow-progressing condition, with half of the patients still alive at 20 years from diagnosis [13]. Younger patients (<60 years) with ET show life expectancy that can extend by up to 33 years. Compared to PV, essential thrombocytosis offers a better prognosis [1]. Despite its indolent nature, patients with essential thrombocytosis experience decreased lifespan compared to the population at large, primarily due to the potential complications of thrombotic events associated with the disease [14]. While the median survival of PMF has improved in the past decade due to increased disease awareness and advancements in treatment options, PMF still exhibits the lowest overall survival among the MPNs, with a reported median survival that reaches 63 months (55–71 months) [15]. In PV, up to 25% of patients experience thrombosis, both arterial and venous, at the time of diagnosis [16]. Following diagnostic assessment, the incidence of non-lethal and lethal thrombotic events during follow-up is reported to be 10% and 5%, respectively [16]. Conversely, a recent meta-analysis in primary myelofibrosis indicated that patients with *CALR* anomaly have fewer thrombotic events. Nonetheless, *CALR*-mutated patients still face an elevated risk of thrombosis compared to individuals without myeloproliferative neoplasms [17]. Heart failure is recognized as a potential complication of myeloproliferative neoplasms (MPNs). The JAK-STAT (Janus Kinase—Signal transducer and activator of transcription) cascade induction, seen in these conditions, has been linked to myocardial fibrosis and restructuring in animal models of hypertensive cardiomyopathy following administration of angiotensin II infusion. Furthermore, current research indicates that the prevalence of pulmonary hypertension ranges from 3% to 7% among MPN patients [18,19]. This condition is correlated with a greater likelihood of heart-related death in MPN patients [20]. Patients diagnosed with primary pulmonary arterial hypertension (PAH) often display elevated levels of circulating proangiogenic precursor cells originating from the bone marrow. Additionally, they may display similar traits with MPNs, such as early reticulin fibrosis, implying a potential common disease mechanism with MPNs [21]. Other possible causes of PAH in MPN patients could imply lung hematopoiesis and sinusoidal obstructive disease [22]. Chronic inflammation is a paramount element in the development of arterial plaque buildup in the general population [23]. In the context of myeloproliferative neoplasms (MPNs), chronic inflammation appears to play a role in initiating and supporting the clone expansion in leukocytes and platelets, which further release proinflammatory mediators [24]. These cytokines, in turn, facilitate blood cell production, perpetuating the cycle loop that exacerbates the risk of premature atherosclerosis in MPN patients [25]. In patients with MPNs, spleen and liver stiffness, evaluated using transient elastography (TE), has been linked to bone marrow fibrosis. Specifically, spleen stiffness is notably higher in PMF and PV compared to ET ultrasound shear wave elastography (SWE) and offers a means to assess tissue stiffness, with point SWE (pSWE) and two-dimensional SWE (2D.SWE) being the most commonly utilized techniques for this purpose [26].

This review aims to compile and analyze recent research examining the relationship between myeloproliferative neoplasms (MPNs) and cardiovascular risk. It focuses on various aspects, including thrombotic risk, atherosclerosis, and vascular rigidity. Additionally, it explores the underlying role of chronic inflammation in these complications. A key aspect of this review is the discussion of emerging research on microRNAs (miRNAs) and their potential role in influencing cardiovascular risk in MPNs. By synthesizing current findings, the review seeks to provide a comprehensive understanding of how MPNs contribute to heightened cardiovascular risk and the mechanisms driving these associations and outlines the future potential of miRNA research in advancing our knowledge in this field.

## 2. Cytokine Dysregulations in MPNs

Inflammation is a vital physiological response that combats invading pathogens, injuries, and toxins by activating the host defense system to promote healing. However, when inflammation becomes prolonged, it becomes long-term inflammation, maintaining the body in constant awareness. This persistent inflammation can lead to genomic instability, which may contribute to the development of neoplasms [27,28]. Both cancerous and healthy cells associated with MPNs produce various inflammatory mediators that significantly contribute to the development of constitutional symptoms. Among these cytokines, Interleukin-1 beta (IL-1β) is a crucial modulator of the inflammatory response [29]. When deregulated, IL-1β is directly linked to MPN progression [30]. This cytokine is released by a few numbers of cells, including monocytes, scavenger cells, and dendritic cells. IL-1β is pivotal in mediating systemic inflammation and stimulates the production of various additional molecules such as IL-6, TNF-α, and colony-stimulating factor (G-CSF), thereby orchestrating the inflammatory response [31,32]. NLRP3 inflammasome genes are upregulated in the hematopoietic cells of MPN patients [33]. Additionally, neutrophil granulocyte-associated lipocalin (NGAL) is often increased in subjects with PV, ET, and PMF in contrast with normal individuals [34]. In a murine model, individuals carrying JAK2-V617F mutation were found to trigger LCN2 activity, leading to DNA strand breaches and apoptosis in adjacent normal cells by generating free radicals and amplifying oxidative stress [35]. Notably, increased plasma levels of TNF-α (tumor necrosis factor-alpha) were significantly associated with the JAK2-V617F allelic load [36]. Numerous studies on MPNs have demonstrated that TNF-α promotes the proliferation of neoplastic cells while inhibiting the growth of normal cells. Reports showed that across different MPNs, including PV, ET, and PMF, high levels of TNF-α were detected [25].

In addition to the increased amounts of proinflammatory mediators, molecules such as IL-4 and IL-10 were also present in higher levels in MPN patients. These anti-inflammatory cytokines are pivotal for mitigating the dysregulated immune state [37].

PMF, PV, and ET each exhibit distinct cytokine signatures that help differentiate these myeloproliferative neoplasms. PMF is defined by increased levels of IL-17, TIMP-1 (Tissue Inhibitor of Metalloproteinases 1), CCL4 (Chemokine (C-C motif) ligand 4), and IGFBP-2 (Insulin-like Growth Factor Binding Protein 2) [38]. Additionally, PMF patients display increased amounts of TNF-α, IP-10, and IL-8 relative to different MPNs, and PMF has the highest overall cytokine production among the three main subtypes of MPNs [39]. PV is distinguished by increased levels of TGF-α (Transforming Growth Factor Alpha) and IL-23 [36,40]. Specific cytokines that are particularly elevated in PV include IL-8, IL-11, leptin, SF (scatter factor), chemokine (C-C motif) ligand 2 (CCL2), IL-10, and IL-22 [41]. Finally, ET is marked by higher levels of eotaxin, EGF (Epidermal Growth Factor), and GRO-α (Growth-Regulated Oncogene Alpha) [36].

The link between clinical presentation and particular inflammatory molecules revealed that elevated IL-8 is linked to general symptoms and increased white blood cells (WBCs). High levels of IL-2R and IL-12 are associated with an increased need for transfusions. SF and MIG (monokine induced by interferon-gamma) are connected to marked splenomegaly [42]. In PV, IL-12 levels were related to HCT levels, IL-1β with WBCs numbers, and IFNα/IFNγ (interferon alpha/gamma) with high platelet count. CCL4 was strongly linked with reduced survival. Additionally, current research revealed that elevated levels of GRO-α were connected to a higher probability of ET conversion to MF (myelofibrosis) [36,43].

## 3. Relation between Inflammation and Thrombosis in MPNs

**Figure 1:**
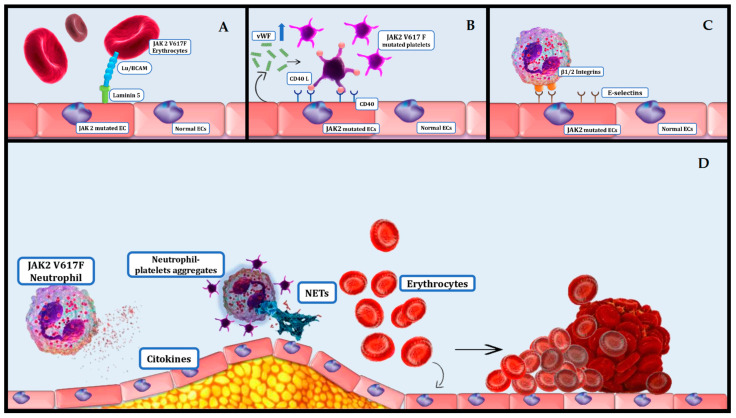
Cardiovascular complications associated with MPNs. (**A**) Lu/BCAM (Lutheran /Basal Cell Adhesion Molecule)-mediated red cell adhesion independently of the erythropoietin receptor (EpoR), utilizing the Rap1/Akt pathway in patients with JAK2 V617F mutation increases thrombotic risk; (**B**) JAK2 V617F mutated endothelial cells (ECs) secrete higher amounts of von Willebrand Factor (vWF), which enhance CD 40 expression on ECs and CD40L on JAK2 V617F platelets increasing platelets adhesion; (**C**) JAK2 V617F mutated granulocytes express abnormal levels of β1/2 integrins that interact with selectins on ECs increasing thrombotic risk; (**D**) JAK2 V617F mutated granulocytes secrete high amounts of cytokines (TNF-α, IL1-β, IL-6 and G-CSF), and there is a high activity of NLPR3 and absent melanoma 2 (AIM2) inflammasome; neutrophil–platelets aggregates are highly adherent to ECs; neutrophil extracellular traps (NETs) are a mixture of DNA, protein, and enzymes that facilitate thrombosis in MPNs.

The overproduction of cytokines leads to the activation of thrombocytes, leukocytes, and endothelial cells. This activation leads to mixed leukocyte–platelet aggregates, which disrupt normal coagulation and cause thrombosis and tissue ischemia (Figure 1D) [44]. Evidence suggests that chronic inflammation may precede the onset of myeloproliferative neoplasms (MPNs), producing a beneficial setting for the extension of the neoplastic cells. Additionally, prior autoimmune conditions are connected to an elevated hazard of MPNs [45]. The JAK2-V617F variant has also been identified in endothelial cells (ECs), where it has been shown that WBCs attach more firmly to JAK2-V617F-mutated ECs than to regular ECs. One study found that numerous genes associated with inflammation and thrombogenic cascades are upregulated in JAK2-V617F-mutated ECs [46,47]. Aberrant secretion of inflammatory cytokines contributes to thrombosis through a variety of cytological processes involving the amplification of VCAM-1 (vascular cell adhesion molecule 1) and ICAM-1 (Intercellular Adhesion Molecule 1) receptors on ECs, activation of integrins and neutrophil enrollment, generation of neutrophil extracellular traps (NETs), thrombocytes activation and aggregation, and tissue factor (TF) release [48]. NF-κB signaling is induced by free radicals, which enhances immune response by promoting the production of proinflammatory cytokines and chemokines [49]. It is known that oxidative stress is more pronounced in patients with ET patients with the JAK2V617F variant who have experienced a thrombotic episode compared to those who have not [50].

A report has shown that 40% of PV subjects suffered a prior thrombotic event upon diagnosis, most of them being arterial in nature [51]. Similar events occur in ET subjects carrying the JAK2-V617F anomaly [52]. Typical events reported in a German study on MPNs were phlebothrombosis, coronary events, strokes, and splanchnic vein thrombosis [53].

The JAK2-V617F mutation induces abnormal activation of leukocyte β-1 and β-2 integrins, which significantly contributes to pathological thrombus formation [54]. Using intra-vital two-photon microscopy, studies have shown that JAK2-V617F-mediated stimulation of neutrophils led to abnormal interactions with the ECs. Remarkably, counteracting β-1 and β-2 integrins reversed the thrombogenic risk in JAK2-V617F transgenic mice [55]. These findings underscore the crucial role of neutrophils in the development of phlebothrombosis induced by JAK2-V617F mutation (Figure 1C) [55].

NETs, consisting of mesh-like components made up of nucleic acids, nucleoproteins, and enzymes combined with antimicrobial proteins that are secreted to trap and kill pathogens, are frequently observed in patients with MPNs. Normally, NETs are pivotal to pathogens defending and inborn immune response, but they also contribute to sterile inflammation [56,57]. During NETosis, neutrophils undergo uncoiling of their DNA, nuclear proteins, and enzyme-like peroxidase into the extracellular environment. NETs have been shown to directly affect platelet function; studies indicate that lipopolysaccharide-stimulated and isolated NETs induce thrombocyte clustering when incubated [58]. These findings highlight the significant role of intrinsic neutrophil activity in promoting thrombosis. Although research on NETs in MPNs remains limited, murine research revealed that elevated JAK2 kinase function correlated with NETs release in MPNs, while targeted therapy significantly reduced thrombogenic risk [59]. The enzyme peptidyl arginine deaminase 4 (PAD4) plays a critical role in chromatin unpacking, disrupting the lobular form of neutrophils and causing nuclear membrane rupture. During both vital and suicidal NETosis, PAD4-mediated histone citrullination is believed to enhance NET formation by promoting chromatin uncoiling and facilitating the release of chromosomal DNA. Therefore, PAD4 is indispensable for NET formation, as demonstrated by studies showing that PAD4-deficient mice are unable to release NETs [60,61]. A study found that patients with MPNs exhibited a relatively increased neutrophil-to-lymphocyte ratio (NLR) compared to normal subjects. This elevated NLR was associated with increased carotid plaque scores, indicating a higher prevalence of carotid artery plaques in MPN individuals. Results like these emphasize the central function of neutrophils in the pathophysiology of MPNs [62].

The inflammatory environment associated with MPNs can lead to increased expression of tissue factor (TF) in monocytes. Elevated levels of cytokines, such as tumor necrosis factor-alpha (TNF-α) and interleukin-6 (IL-6), are commonly observed in MPNs and can stimulate monocytes to upregulate TF expression [63]. In vitro experiments using lipopolysaccharide (LPS) stimulation demonstrated a significant increase in TF expression on monocytes from all study subjects compared to non-stimulated monocytes (*p* < 0.05 for all groups). Notably, monocytes from patients with PV and ET showed higher levels of TF expression compared to control subjects [64]. Clonal platelets and micro-vesicles release excessive amounts of tissue factor (TF) following tissue and vascular damage. This excessive TF release activates the coagulation cascade, leading to increased thrombin generation. Thrombin acts as a protease that activates protease-activated receptors (PARs) on platelets and other cells. The activation of PARs has several key effects: it facilitates the conversion of fibrinogen into fibrin by diminishing the binding sites on platelets (PFR), promotes vasoconstriction, and enhances platelet activation. Additionally, thrombin-induced signaling interacts with the TGF-beta pathway, which contributes to inflammation and fibrosis [65,66].

## 4. Platelets and Erythrocytes Effect on Thrombosis in MPNs

Thrombocytes still play an equivocal role in thrombogenic risk linked with JAK2-V617F positive MPNs. Studies on transgenic mice, which examined platelet involvement in different thrombosis models, have yielded mixed findings. In ET, the abnormal function of the PI3 kinase/Rap1/integrin *α*IIb*β*3 cascade has been linked to low thrombocyte reactiveness [67]. Additionally, thrombocytes from CALR ET subjects exhibit reduced reactivity when stimulated with ADP (adenosine diphosphate) in contrast to normal subjects or JAK2 variants of ET [68]. There is significant debate surrounding the function of high platelets count in thrombogenicity since no association between elevated platelets and thrombotic accidents has been shown [69]. In fact, platelets with counts exceeding high levels (>1500 × 10^9^/L) are more prone to hemorrhagic events rather than thrombotic accidents. In subjects with ET experiencing extreme thrombocytosis, leukocytosis enhances the thrombotic risk [70]. However, cytoreductive therapy aimed at reducing platelet counts has been shown to help prevent both thrombotic and bleeding complications [71,72]. Current guidelines recommend maintaining platelet counts below 400 × 10^9^/L to mitigate these risks [73].

In the prothrombotic context of MPNs, platelet interactions with leukocytes are critical. Thrombocytes attach to granulocytes through proteins like P-selectin that encounter its ligand PSGL-1, GPIIa, and GPIIbIIIa (by fibrinogen), each of them interacting with CD11b/CD18 (Mac-1). Higher amounts of P-selectin are directly connected with high thrombocytes–granulocytes clusters in MPNs [74,75,76]. This platelet–leukocyte interaction activates both cell types, contributing to a heightened prothrombotic state. Endothelial activation in MPNs is well-documented, indicated by increased levels of vWF (von Willebrand factor), thrombomodulin, and selectins [77,78]. Stimulated ECs display prothrombotic traits, enrolling platelets and leucocytes. The discharge of vWF leads to platelet activation, causing CD40 ligand (CD40L) to surface on platelets and bind to endothelial CD40. Cleaved CD40L forms a dissociated fragment (sCD40L), often elevated in MPN subjects (Figure 1B) [78]. Interestingly, the JAK2V617F variant was detected in adult ECs from certain structures, including the spleen of patients with PMF and the liver of PV patients with Budd–Chiari syndrome. This suggests a possible connection of ECs with the neoplastic cells [79,80]. ET patients exhibit numerous streaming microparticles derived from platelets, ECs, and leukocytes, the former being more prevalent. These microparticles are abundant with tissue factors and carry platelet-related mediators, intensifying the inflammatory and prothrombotic potential of platelets [81].

Thrombosis and cardiovascular complications are notably more prevalent in PV compared to other myeloproliferative disorders. Studies indicate that nearly 40% of PV patients experience either minor or major thrombotic events, with vascular-related deaths accounting for 35% to 45% of all fatalities in PV cases [82]. Accurately assessing the thrombotic risk in PV is critical for making informed treatment decisions. PV patients are typically stratified into two risk groups: high-risk (patients aged over 60 years or with a history of previous thrombosis) and low-risk (patients without these risk factors) [82].

In one study, hypertension was identified as a significant predictor of overall thrombosis, major thrombosis, and arterial thrombosis in patients with MPNs treated with anagrelide. However, hypertension did not predict microvascular or venous thrombotic events based on univariate analysis. In multivariate regression analysis, hypertension emerged as the strongest predictor of arterial thrombotic events, with an odds ratio of 1.813 (95% CI 1.295–2.538, *p* = 0.001) [83]. Another study also investigated the relationship between cardiovascular risk factors (CVRFs) and thrombosis in MPN patients. Among 403 MPN patients (165 with PV and 238 with ET), hypertension was the most prevalent cardiovascular comorbidity, affecting approximately 64% of individuals in both subgroups [84].

Drug-induced hypertension is frequently underrecognized in clinical practice. For instance, in the RESPONSE-2 trial, 9% (n = 7) of the 74 PV patients receiving ruxolitinib experienced hypertension as a non-hematological adverse event. Among these, hypertension was more commonly observed as a grade 3–4 adverse event (grade 3: 5%, n = 4; grade 4: 1%, n = 1) compared to grades 1–2 (3%, n = 2). In contrast, PV patients receiving the best available treatment experienced hypertension only as a grade 3 adverse event (4%, n = 3) [85].

One of the primary criteria for diagnosing polycythemia vera (PV) is the occurrence of increased hematocrit (HCT) due to raised numbers of erythrocytes. The thrombogenicity of high HCT in PV is well documented [86,87]. Studies indicate that subjects who uphold an HCT lower than 45% are less prone to vascular events, in contrast with individuals with an HCT maintained between 45% and 50%. The plasticity of red blood cells (RBCs) is crucial for blood flow dynamic, and even a small decline can significantly heighten vascular stream resistance and blood viscosity, potentially leading to clot formation. Research using laser-assisted techniques to study PV RBC morphology has demonstrated a notable reduction in RBC deformability [88]. Furthermore, elevated activity levels of glucose-6-phosphate dehydrogenase and acetylcholinesterase in PV RBCs have been documented. These findings, along with elevated levels of glutathione and malondialdehyde, may contribute to the increased rigidity observed in PV RBCs [89]. Regarding the thrombogenic potential of elevated HCT, evidence shows MPNs surpass secondary causes such as lung conditions. Research has demonstrated that the JAK2-V617F mutation can activate Lu/BCAM-mediated red cell adhesion independently of the erythropoietin receptor (EpoR), utilizing the Rap1/Akt pathway. This pathway is believed to be pivotal to the atypical interplay between blood cells and ECs in PV individuals, potentially contributing to thrombotic events (Figure 1A) [90].

Another contribution to thrombosis is Lysyl Oxidase (LOX), an enzyme released in the interstitium, which plays a crucial role in collagen and elastin fibril maturation by facilitating crosslink formation [91]. Recent evidence supports the existence of high serum concentrations of LOX in MPN individuals [92]. Beyond its role in collagen crosslinking, recent findings associate LOX with MPN evolution, especially PMF and thrombogenic. Increased LOX levels have been shown to enhance platelet–collagen interaction, leading to their activation [93,94]. Additionally, this enzyme may play a role in cardiac comorbidities via platelet-derived growth factor (PDGF) receptor oxidation, which, in turn, could lead to a higher affinity of PDGF to its receptor, reducing the clearance of molecules involved in the PDGFR signaling cascade, enhancing proliferation and fibrosis. Furthermore, LOX may increase extracellular matrix stiffness, thereby impacting vascular integrity and function [95].

## 5. Complementary Role of JAK 2 Variants and Clonal Hematopoiesis of Indeterminate Potential (CHIP) in Cardiovascular Risk

JAK2 belongs to cytoplasmic tyrosine kinase family members responsible for mediating signal transduction from cell surface cytokine receptors like erythropoietin and thrombopoietin receptors [96]. Upon ligand binding to the receptor, JAK undergoes autophosphorylation and transphosphorylates the receptor, creating binding sites for signaling molecules such as STAT proteins. Phosphorylated STATs then translocate to the nucleus to influence target gene transcription. For instance, the interaction of erythropoietin with its receptor (EpoR) triggers the phosphorylation of JAK2 and STAT5, as well as the activation of other effectors, including the MAP kinase and PI3K/Akt pathways [97,98]. Patients with myeloproliferative neoplasms (MPNs) often carry additional mutations associated with heightened inflammation, including those in the TET2, DNMT3a, and ASXL1 genes [99]. While individuals with CHIP, in most cases, might not acquire hematological malignancies, they are most likely susceptible to cardiac comorbidities and enhanced arterial plaque buildup [100]. The association with JAK 2 variants is particularly concerning, as it poses a maximum risk for coronary syndrome, as evidence shows that this scenario counts for 20% of the patients with CHIP and myocardial infarction [100]. In young adults, JAK2 variants nearly quadruple the risk of myocardial infarction. Furthermore, evidence suggests that CHIP is a deciding factor regarding overall outcomes in individuals with heart failure [101]. Another study demonstrated that CHIP significantly increased cardiovascular disease risk, with the highest risk associated with the JAK2V617F mutation. Neutrophils from both mice and humans with the JAK2V617F mutation are more prone to undergoing NETosis compared to wild-type cells, suggesting that JAK2V617F may exacerbate conditions like superficial erosion by promoting NETosis [102].

Patients with CHIP, characterized by anomalies in DNMT3A, TET2, ASXL1, or JAK2 genes, are susceptible to inflammatory-associated conditions like ischemic cardiac disease. CHIP is considered to precede MDS and reflects the presence of mutations that are also implicated in MDS pathogenesis, thus linking these genetic alterations to inflammatory and cardiovascular outcomes [103,104].

## 6. Impact of MPNs on Atherosclerosis

Plaque formation is a dynamic process influenced by various mechanisms, including cholesterol expulsion, that could alleviate or potentially reverse plaque development [105,106]. This process is clinically significant in reducing cardiovascular events. The initial step of cholesterol expulsion requires the efflux of cholesterol from the arterial wall into the liver initiated by ApoA1 (apolipoprotein A1) attachment to ABCA1 (ATP-binding cassette transporter A1) on foam cells, facilitating cholesterol efflux and HDL (high-density lipoprotein) expansion. The latter can also interact with various receptors that could lead to further cholesterol expulsion. Cholesterol is then either transported to ApoB or straight to the liver through scavenger receptors (SRB1) [106,107].

In a study involving LDL-R, knocked-out mice infused with medullary aspirate from JAK V617F positive mice with increased atherosclerosis with greater and unstable plaques were observed [108]. JAK 2 V617F variant was associated with higher activity of the AIM2 (absent in melanoma 2) signaling pathway and IL1-β. Blockade of the latter mediator could decrease necrotic core extension and fibrous plaque shrinking in various experimental atherosclerosis studies on mice [109]. Most patients with PV harbor the JAK2V617F mutation. Macrophages with this mutation exhibit impaired efferocytosis, leading to enlarged necrotic cores. Additionally, JAK2V617F-mutated macrophages display enhanced phagocytosis of RBCs in contrast to normal subjects, which leads to iron discharge from hemoglobin disintegration and release of free radicals [110,111]. Furthermore, JAK2 deficiency has been shown to accelerate atherosclerosis primarily as a result of deficient cholesterol export. Results like these have important therapeutic consequences for patients with MPNs with prolonged JAK-STAT signaling blockade since this is linked to an elevated cardiovascular risk. Therefore, careful clinical assessment is essential for managing cardiovascular risk in these patients, as it may be adversely affected by JAK2 inhibition [112].

One research examined the evolution of carotid artery rigidity between JAK2 mutated ET subjects and regular patients. The findings revealed that those with the JAK2 mutation experienced a more rapid increase in carotid artery stiffness, which served as a surrogate marker of arterial plaque buildup relative to normal individuals [113]. On the other hand, another study assessing carotid arterial stiffness and digital endothelial function found no significant differences between ET subjects and normal patients regarding these features. Additionally, the same report revealed that coronary calcium burden was relatively higher in ET patients, which could contribute to cardiac events, an aspect that was not estimated by the Framingham risk score [23]. Abdominal aortic calcification (AAC) is prevalent in ET patients and is linked to arterial thrombotic events [114].

Myeloproliferative neoplasms may induce distal embolism from unstable atherosclerotic plaques by promoting a prothrombotic condition. These distal ischemic events were often the initial indication of ET, leading to its diagnosis. Notably, in ET, thrombotic events can occur even when the platelet count is normal. This was observed in cases of foot ischemia, where microvascular thrombosis associated with ET coincided with a proximal ulcerating atherosclerotic plaque [115].

Beyond inflammatory cytokines, various molecules like TGF-β (transforming growth factor) and several others are likely involved in promoting atherothrombosis in MPNs [37]. Studies have shown that MPNs are linked to higher TGF-β concentrations in plasma [116]. Increased TGF-β signaling in ECs has been linked with coronary injury in patients with cardiac ischemic disease [117]. Moreover, a report on peritoneal dialysis showed that higher plasma concentrations of the same molecule were related to the carotid intima-media ratio [118]. Although elevated TGF-β in MPNs promotes arterial plaque expansion, the function of this molecule is not clearly defined, making it a promising area of research in perspective [99]. TGF-β also plays a role in cell phenotype switch, an event contributing to oncogenesis and, potentially, to both atherogenesis and fibrosis within the bone marrow niche [119]. Recent studies underscore the pivotal roles of bone marrow precursors, stem cells, and immune cells in cardiovascular disease (CVD) initiation and progression. Genetic analyses have revealed that clonal hematopoiesis, marked by proliferation of bone marrow progenitor and stem cells with somatic mutations, is prevalent in about 10% of the general population, increasing with age. This condition, which is distinct from the cumulative risk of blood cancers, is now recognized as a significant risk factor for CVD. Among the most critical mutations in clonal hematopoiesis are JAK2V617F and Tet methylcytosine dioxygenase 2 (TET2) [120]. These mutations in hematopoietic cell clones are linked to the pathogenesis of CVD. These findings highlight the significant role of CHIP in association with MPNs [120].

## 7. Additional Factors Contributing to Cardiovascular Risk in MPNs

The role of systemic renin–angiotensin system (RAS) induction in arterial plaque formation among MPN subjects is still presumptive and requires supplementary research [99]. Inappropriate RAS activation has numerous harmful effects, including promoting atherosclerosis, causing endothelial damage, inducing insulin resistance, and having prothrombotic effects. It also stimulates vascular smooth muscle cell and monocyte proliferation. The RAS exacerbates coronary heart disease (CHD) progression through angiotensin-II, which impacts vascular cells both directly and indirectly by increasing free radicals production and decreasing nitric oxide (NO) in ECs [121]. Intensified RAS function could be a consequence of heightened JAK/STAT signaling in precursor and stem cells from the hematogenic marrow of MPN individuals [99]. Studies have found that PV and ET exhibited distinct patterns of major RAS components in bone marrow compared to normal bone marrow, closely linked to the JAK2V617F mutation rather than the disease type. Campbell et al. categorized ET into two subtypes based on the JAK2 mutation, with the JAK2V617F-positive subtype resembling PV phenotypically [122]. This finding was supported by the similarity in expression patterns of major RAS components between JAK2V617F-positive ET and PV groups [123]. Additionally, ACE (angiotensin-converting enzyme) blockade in PV individuals optimized HCT levels and reduced the reliance on cell-reducing therapy [124]. In experimental myelofibrosis models in mice, ACE inhibitor administration led to spleen size reduction and alleviated fibrous transformation of bone marrow [125].

Pulmonary hypertension (PAH) and right ventricular impairment are known complications of MPNs, although the prevalence of the former within this patient group remains poorly characterized [126,127]. The numerous hemangioblasts present in the bloodstream might be an important factor in PAH pathophysiology in MPN subjects [128]. Studies indicate that hemangioblasts, identifiable in adult bone marrow by their CD133 expression on a subset of CD34-positive hematopoietic stem cells, play significant roles in blood cell formation [129,130]. While their contribution to postnatal endothelial development remains uncertain, CD34+CD133+ progenitors are recognized for their proangiogenic effects on endothelial cells during angiogenesis [131]. Additionally, elevated angioblasts in the bloodstream are a common finding in Primary PAH individuals. Furthermore, these patients also exhibit MPN-like traits such as reticulin fibrosis in the bone marrow, indicating a potential common disease mechanism [21,132]. Persistent thromboembolic pulmonary hypertension is rarely found in MPN subjects, and it is most likely a consequence of reoccurring emboli and other possible genetic factors [133].

## 8. Other Biological Markers Associated with Cardiovascular Disease in MPNs

MicroRNAs play crucial roles in human diseases, including cardiovascular conditions. MicroRNA-155, a versatile molecule, is involved in blood precursor cells maturing, immune response, and vessel restructuring, and it is related to conditions like ischemic heart disease, heart failure, and cardiac complications of diabetes. However, its role in atherosclerosis is complex, with studies showing that it can both promote and inhibit the disease, highlighting its multifaceted nature [134,135]. In a study, an inverse relationship was identified between the numbers of miR-155 transcripts and certain gene expression.

miR-221 and miR-203 were highly expressed in JAK2V617F negative ET subjects and act as ligands for SOCS 1 (Suppressor of Cytokine Signaling 1) and SOCS 3 proteins, respectively. These proteins act as modulators for different pathways, reducing JAK/STAT cascade activity with immunological and proliferative consequences [136,137].

miR-28 acts as a regulator of the thrombopoietin receptor MPL by inhibiting its translation, leading to a reduction in thrombocyte count. Research has indicated a connection between miR-28 and wild-type ET. This link suggests that JAK-STAT pathway activation might lower miR-28 levels, which could explain the higher thrombocyte counts observed in wild-type JAK2 ET patients [122,138].

A recent investigation of peripheral blood CD34+ cells in PV patients found that miR-196b is downregulated in both male and female patients. This deregulation of stem-progenitor cell miRNAs might play a role in the clonal expansion of JAK2V617F. Continued research in this area could provide deeper insights into MPN pathogenesis and help identify potential targets for therapies aimed at eradicating the disease [139].

Glycoprotein YKL-40 is released by different cells during the inflammatory process, such as neutrophils and macrophages, as well as cancerous cells. In the bone marrow of healthy individuals, this molecule is deposited in the vesicles of precursor granulocytes [140,141,142,143,144]. Emerging evidence suggests that YKL-40 may promote atherosclerosis by contributing to endothelial dysfunction through different mechanisms, with the most important being endothelial injury-induced organ restructuring. Furthermore, YKL-40 is overexpressed in scavenger cells and myocytes within arterial plaques [145]. MPN subjects displayed relatively higher concentrations of plasma YKL-40 compared with healthy controls and were indicative of a high inflammatory state, reduced functional status, general symptoms, and cardiovascular disease. In MF patients, higher concentrations of YKL-40 were related to anemia degree, presence of blasts, and clinical scores [146].

Table 1 summarizes the information about myeloproliferative neoplasms and the association with cardiovascular risk and complications.

## 9. Conclusions

This study highlights the substantial cardiovascular risks in MPNs, primarily driven by genetic mutations and chronic inflammation. Mutations in JAK2, MPL, and CALR genes play crucial roles in disease progression and thrombotic complications, with PV and ET showing high thrombosis rates. In PMF, the CALR variant predicts a lower thrombotic risk relative to JAK2 mutations, but the risk remains elevated relative to the general population.

Chronic inflammation worsens cardiovascular complications by promoting clonal expansion and increasing leukocyte and platelet production. Heart failure and pulmonary hypertension linked to JAK-STAT pathway activation add to the cardiovascular burden in MPN patients.

## Figures and Tables

**Table 1:** International Consensus Classification 2022 for myeloproliferative neoplasms and associated cardiovascular risk factors and complications.

MPNs	Major Criteria	Minor Criteria	Cardiovascular Risk Factors and Complications	References
PV	hemoglobin: >16.5 g/dL in men; >16.0 g/dL in women; hematocrit: >49% in men and >48% in women JAK2 V617F or JAK2 exon 12 mutation	EPO concentrations lower than normal	Hypertension (40–70%) [147] Diabetes melitius (7–16%) [148] Obesity (7.5%) and smoking (5–10%) [38] Myocardial infarction (30%) [149] Stroke (25%) [149] Peripheral arterial thrombosis (20%) [149]	[38,147,148,149,150,151]
ET	Platelet count ≥ 450 × 10^9^/L JAK2, CALR, or MPL mutation	Exclusion of secondary causes of platelet number increase	Thrombotic risk before diagnostic 18% [152] Each cardiovascular risk increases the thrombotic risk [153]: No CV risk (14%) 1 CV risk (16%) More than 1 CV risk (29%)	[150,151,152,153]
PMF	High-grade reticulin fibrosis (2 or 3 grade) JAK2, CALR, or MPL mutation	Secondary anemia excluded Leukocytosis ≥ 11 × 10^9^/L Enlarged spleen LDH levels raised above normal interval Leucoerythroblastosis	Thrombotic events [154]: Myocardial infarction (9.5%) Stroke (2.3%) Venous thromboembolism (4.5%) Splanchnic thrombosis (0.6%)	[150,151,154]

JAK2—Janus kinase 2; MPL—myeloproliferative leukemia protein; CALR—calreticulin; EPO—erythropoietin; LDH–lactate dehydrogenase.
